# An Evaluation of Characters for the Separation of Two *Culex* Species (Diptera: Culicidae) Based on Material From the Upper Midwest

**DOI:** 10.1093/jisesa/ieaa119

**Published:** 2020-11-04

**Authors:** Lílian Ferreira-de-Freitas, Nicholas B Thrun, Bradley J Tucker, Lauren Melidosian, Lyric C Bartholomay

**Affiliations:** 1 Pathobiological Sciences Department, University of Wisconsin–Madison, Madison, WI, USA; 2 Departamento de Zoologia, Universidade Federal do Paraná, Avenida Coronel Francisco H. dos Santos, Jardim das Américas, Curitiba, PR, Brazil

**Keywords:** *Culex pipiens* s.l, *Culex restuans*, morphology, character reappraisal, occipital erect scales

## Abstract

Mosquitoes (Diptera: Culicidae) in the *Culex pipiens* complex play a key role in the transmission and therefore epidemiology of a number of human and animal pathogens globally. These mosquitoes, and sympatric species of the genus *Culex* Linnaeus that are not within the *Cx. pipiens* complex, are often considered ‘impossible’ to distinguish by morphology in the adult female stage. In the United States, this is particularly true for *Culex pipiens* s.l. and *Culex restuans* Theobald, both of which are competent vectors of West Nile virus, but likely play different roles in the transmission cycle. Therefore, we undertook an in-depth morphological evaluation of matched larval exuviae and adult specimens that revealed five useful morphological characters that are informative to distinguish *Cx. pipiens* s.l. from *Cx. restuans* in the adult stage. Herein, we provide a comprehensive review of the literature on these species of interest, and four additional, morphologically similar, *Culex* species, and a proposed key to adult female specimens.

*Culex pipiens* Linnaeus is the namebearing taxon for the family Culicidae (Order Diptera) and is a member of one of the most taxonomically challenging complexes of species of mosquitoes. In the United States, the *Culex pipiens* complex is represented by *Culex pipiens* Linnaeus and *Culex quinquefasciatus* Say, as well as hybrid forms of those two (Smith and Fonseca 2004, Farajollahi *et al.* 2011). These mosquitoes, and sympatric *Culex* species that are not within the *Cx. pipiens* complex, are often considered impossible to distinguish by morphology in the adult female stage. In the United States, this is particularly true for *Culex pipiens* s.l. and *Culex restuans* Theobald.

*Culex pipiens* and other *Culex* species transmit a number of neuroinvasive arthropod-borne viruses including Western equine encephalitis virus, St. Louis encephalitis virus, and West Nile virus (WNV). In particular, in Eastern and Midwestern states, members of the *Cx. pipiens* complex and *Cx. restuans* are key players in the transmission of WNV (Johnson *et al.* 2015). Because of their vector status, adult female *Cx. pipiens* complex and *Cx. restuans* are regularly targeted in arbovirus surveillance efforts. Unfortunately, the morphological traits used to separate adult female members of the *Culex pipiens* complex and *Culex restuans* Theobald present in current keys are fraught with ambiguity; indeed, many authors have chosen to not differentiate between these taxa in their publications, as most collections consist of adult females (e.g., Michener 1947, Meece *et al.* 2003, Kilpatrick *et al.* 2005, Kinsley *et al.* 2016).

This course of action is understandable given the morphological similarity of females of these species, but is problematic for the study of the particular contribution of each of these species to WNV enzootic, epizootic, endemic, or epidemic transmission cycles. In the Upper Midwestern states, both species feed on avian hosts (Hamer et al. 2008). However, some authors speculate that *Cx. pipiens* s.l. may feed on mammals more than previously thought (Farajollahi *et al.* 2011) and possibly more than *Cx. restuans* (Apperson *et al.* 2002). As such, *Cx. restuans* may be less likely to transmit WNV to humans, but may be critical for early season amplification of the virus in the enzootic cycle (Johnson *et al.* 2015). It is important, then, to be able to differentiate between these taxa in order to further evaluate the relative importance of each species to WNV transmission to humans in the United States.

Although it is possible to distinguish these species using a PCR diagnostic, morphological identification would facilitate rapid processing of field-collected mosquitoes. Therefore, we aimed to develop accurate morphological diagnostic criteria for these two *Culex* species. Towards this goal, we reviewed characters used in North American keys as well as all characters described for both taxa in the literature. Here we provide a comprehensive discussion of the use and utility of characters represented in keys and faunistic reviews for the mosquitoes of North America (see Background). Importantly, we also report that four other *Culex* species were poorly characterized in certain keys and other reference type literature, and specimens of these six species could be misidentified as one another if based only in said characters, so we included those extra four species in our analysis.

We performed an in-depth analysis of distinct character states between *Cx. pipiens* s.l. and *Cx. restuans* adult females, and report five characters that may be useful in the differentiation of these two species. The utility of these characters for separating *Cx. pipiens* s.l. and *Cx. restuans* was validated using the PCR diagnostic for *Cx. pipiens*, *Cx. restuans*, and *Culex salinarius* Coquillett as described by Crabtree *et al.* (1995). The molecular diagnostic confirmed the morphological identification for every specimen tested from Wisconsin, Illinois, and Minnesota. Finally, a provisional key is presented for six morphologically, superficially similar *Culex* species, including both *Cx. pipiens* s.l. and *Cx. restuans*.

## Background

The characters that are used in key couplets to separate adult female *Cx. restuans* and *Cx. pipiens* s.l. from one another and other morphologically similar species are presented here for each of the published keys to mosquitoes of North America, i.e., Ross (1947), Carpenter and LaCasse (1955/1974), Wood *et al.* (1979), Means (1987), Darsie and Ward (2005), and Andreadis *et al.* (2005).

### Characters in Common Use to Separate *Cx. pipiens* s.l and *Cx. restuans*

The most commonly used character to separate *Cx. restuans* from *Cx. pipiens* s.l. up until now has been the middorsocentral patches of pale scales on the scutum (Ross 1947, Carpenter and LaCasse 1955/1974, Wood *et al.* 1979, Means 1987, Andreadis *et al.* 2005). Unfortunately, it is also clear from the literature that this character is unreliable because a portion of *Cx. restuans* in any given sample will simply not present the phenotype. Indeed, Wood *et al.* (1979) used the qualifier ‘usually’ to warn the reader that the spots might not be present. The proportion of specimens that exhibit the character in a given population varies greatly from place to place and possibly seasonally, making it impossible to generalize and/or predict the occurrence of variation.

The second most used character (and perhaps the one portrayed by keys as the most reliable choice) is the narrowing basal ‘pale banding’ towards the edges of the abdominal terga, which Ross used to separate *Cx. quinquefasciatus* from *Cx. pipiens* + *Cx. restuans* (1947). Indeed this character separates *Cx. pipiens* from *Cx. restuans* according to Carpenter and LaCasse (1955/1974), Means (1987), Andreadis (2005), and Darsie and Ward (2005).

### Characters Used to Distinguish Morphologically Similar Species

Morphological similarities similarly confound the identification of *Cx. erythrothorax* Dyar, *Cx. interrogator* Dyar & Knab, *Cx. chidesteri* Dyar, *Cx. salinarius* Coquillett, and *Cx. pipiens* s.l. and *Cx. restuans* Theobald. In the analysis below key characters are presented that can be used to distinguish these species in the context of the body regions for which diagnostic characters have been reported.

### Characters on the Scutum

Ross separated *Cx. restuans* from *Cx. pipiens* by the middorsocentral pale scaling on the scutum (1947). Carpenter and LaCasse (1955/1974) separated *Cx. interrogator* from *Cx. restuans* by the lack or usually presence of middorsocentral spots of pale scaling on the scutum, respectively. Means and Andreadis et al. also mentioned the existence of a patch of middorsocentral pale scales on the scutum of *Cx. restuans* as diagnostic for the species (1987, 2005).

Carpenter and LaCasse (1955/1974) considered the scaling on the scutum to be ‘somewhat coarse’ and ‘golden’ for *Cx. pipiens* s.l., and ‘fine’ and ‘golden brown’ for *Cx. interrogator* + *Cx. restuans*. Means also separated *Cx. restuans* from *Cx. pipiens* based on the character of ‘fine scaling’ vesus ‘coarse scaling’, respectively (1987). Apperson *et al.* (2002) explained the character in more detail stating the scutal scales are typically longer, falcate and lighter brown on *Cx. pipiens,* and shorter, not falcate and darker brown for *Cx. restuans*. The result is an appearance that the scaling is ‘lighter, coarse and scruffy’ in *Cx. pipiens*, and ‘darker, smooth and *well arranged*’ in *Cx. restuans*.

### Characters on the Scutellum

Apperson et al. and subsequently Andreadis et al. used the character of the scutellum of *Cx. salinarius* as having short brown scales, whereas *Cx. pipiens* + *Cx. restuans* have long pale scales (2002, 2005).

### Characters on the Lateral Thorax

Carpenter and LaCasse (1955/1974) separated *Cx. nigripalpus* Theobald from *Cx. salinarius* + *Culex chidesteri* Dyar by the pleural scaling pattern. The authors did not provide a clear explanation of this character, but Belkin et al. (1970) stated that the pleural pale scaling of *Cx. nigripalpus* is restricted to a few broad pale scales on the upper and lower mesokatepisternum and a few narrow pale scales on the upper mesepimeron. They treated the pleural scale patches of *Cx. chidesteri* as equal to that of *Cx. quinquefasciatus*, other than presenting a few postspiracular scales, i.e., a small patch of broad pale scales on upper proepisternum, broad pale scales on the upper and lower mesokatepisternum, a large patch of elongate broad pale scales on the mid mesepimeron, and many elongate pale scales on the upper mesepimeron. To the best of our knowledge, the pattern of pleural scaling of *Cx. salinarius* has not been described by any author to date.

### Characters on the Tibia and Tarsi

Carpenter and LaCasse (1955/1974) differentiated *Cx. chidesteri* from *Cx. nigripalpus* with the description that all of the tibiae in *Cx. chidesteri* have more or less conspicuous ‘knee spots’ of pale scales as well as extremely narrow rings of pale scaling at the tarsal joints whereas *Cx. nigripalpus* has inconspicuous ‘knee spots’, except sometimes on hind leg, and all tarsi dark. Means (1987) also considered the tibial ‘knee spots’ of *Cx. restuans* as ‘prominent’ while the ones on *Cx. pipiens* ‘moderate’.

### Characters on the Abdominal Tergites

Ross (1947) stated that *Culex salinarius* can be distinguished from *Cx. restuans* + *Cx. pipiens* s.l. by presentation very narrow ‘pale banding’ on all or most of the otherwise brown scaled tergites. He further separated *Cx. quinquefasciatus* from *Cx. restuans* + *Cx. pipiens* based on the same character forming narrowing ‘pale banding’ towards the edges of the segments. Carpenter and LaCasse (1955/1974) likewise separated these *Culex* species according to those that have broader ‘basal banding’ on the abdominal tergites (*Cx. pipiens* s.l. + *Cx. interrogator* + *Cx. restuans*) from the species that have narrow to nonexistent basal ‘pale banding’ (*Culex nigripalpus* + *Cx. salinarius* + *Cx. chidesteri*). Means also separated *Cx. salinarius* from *Cx. restuans* + *Cx. pipiens* by the presence of the very narrow ‘pale banding’, often absent, adding that segments VII and VIII are mostly covered by pale yellow scales and uses the character of narrowing basal ‘pale banding’ of the tergites to separate *Cx. pipiens* from *Cx. restuans* (1987). Andreadis et al. also used the character of the tergite basal ‘pale bands’ narrowing towards the edges of the segment in *Cx. pipiens* to separate it from *Cx. restuans* (2005).

Carpenter and LaCasse also separated *Cx. salinarius* from *Cx. chidesteri* by contrasting abdominal tergite scaling. They reported pale yellow scaling scattered near the apex of each segment and tergite VII mainly or entirely covered by pale yellow scales for *Cx. salinarius*. For *Cx. chidesteri,* the tergites have only dark scaling at apex, with tergite VII mainly covered by dark scaling (1955/1974).

## Evaluation of Characters Using Field-Caught Specimens

Apperson *et al.* (2002) used the following characters to differentiate *Cx. salinarius*, *Cx. pipiens* s.l., and *Cx. restuans* specimens collected in the Borough of Queens, New York City: 1) the presence of short brown scales on the mid lobe of the scutellum of *Cx. salinarius*, in contrast to these scales long and pale (cream or white, in their words) in *Cx. pipiens* s.l. and *Cx. restuans*; 2) the sternites entirely covered by ‘copper’ scales in *Cx. salinarius*; 3) the ‘dingy yellow to copper colored’ scales forming the basal ‘bands’ of the tergites, these very narrow to absent on II and III, in contrast with the same character as yellow or ‘cream’ colored and appearing ‘convex’ for *Cx. pipiens* s.l. (narrowing towards edges), whereas those from *Cx. restuans* are white colored and appearing broad and connected with the basolateral patch. The authors noted that some character overlap exists with *Cx. pipiens* s.l. and *Cx. restuans*; 4) the ‘dingy yellow to copper colored’ scales covering tergites VII and VIII, presumably in contrast with the scaling pattern of tergites VII and VIII not different from other tergites in *Cx. pipiens* s.l. and *Cx. restuans*; 5) ‘reddish-brown’ coloring of the thoracic integument in *Cx. restuans*, in contrast with the ‘tan to brown’ coloring in *Cx. pipiens* s.l.; the scales of the scutum ‘dark brown’, short and not falcate in *Cx. restuans*, in contrast to ‘light brown’, long and falcate in *Cx. pipiens* s.l. The authors report a success rate of identification in using this combination of characters of 100% for *Cx. pipiens*, 100% of *Cx. salinarius*, and 80% for *Cx. restuans*, for which the remaining (20%) of misidentified specimens were *Cx. pipiens* (Apperson *et al.* 2002). Their morphological identifications were confirmed using the technique described by Crabtree *et al.* (1995).

Harrington and Poulson reevaluated the characters presented by Apperson et al. to differentiate *Cx. pipiens* s.l. from *Cx. restuans* from central New York (2008). They report the combination of characters would correctly identify only ~60% of *Cx. pipiens* s.l. and ~30% of *Cx. restuans*, based on results from PCR diagnostics designed by Crabtree *et al.* (1995). They concluded that these mosquito species cannot be conclusively identified by morphological characters used by Apperson *et al.* (2002).

McKinnish *et al.* (2013) evaluated specimens from Pennsylvania and Virginia collected with gravid traps and confirmed morphological identification using the PCR diagnostic designed by Crabtree *et al.* (1995). Only 31% of their *Cx. restuans* specimens had middorsocentral pale scaling spots on the scutum. They also found the difference in abdominal banding pattern from ‘straight’ bands in *Cx. restuans* to ‘curved’ bands in *Cx. pipiens* s.l. could be used to correctly identify 87% of *Cx. restuans* and 84% of *Cx. pipiens* s.l. Interestingly, they also evaluated the presence of pale erect scales on the head for *Cx. pipiens* s.l. in contrast with the presence of only dark erect scales for *Cx. restuans* and report the character correctly identified 92% of *Cx. pipiens* s.l. and 93% of *Cx. restuans*. They concluded the combination of these characters should be diagnostic for these species.

## Materials and Methods

### Molecular Methods

Genomic DNA was extracted from a single mosquito leg utilizing the protocol described by Johnston *et al*. (2015), where the mosquito leg is placed in a tube with 50 µl of 25 mM NaOH + 0.2 mM EDTA at pH 12, which is heated to 95°C for 1 h then cooled down to 4°C and 50 µl of 40 mM EDTA at pH 5 is added.

In accordance with previous literature, the primers used for molecular identification were designed by Crabtree *et al.* (1995) for the ITS regions of rDNA, with the following sequences (shown 5′ to 3′): reverse primer, (CP16) GCGGGTAC CATGCTTAAATTTAGGGGGTA; forward primer for *Cx. pipiens* s.l. (PQ10) CCTATGTCCGCGTATACTA; forward primer for *Cx. restuans* (R6) CCAAACACCGGTACCCAA; forward primer for *Cx. salinarius* (S20) TGAGAATACATACCACTGCT. The PCR mixture for each tube consisted of 1 µl of extraction product, 1.5 µl of each primer (CP16, PQ10, R6, S20) at a concentration of 10 μM, 5 µl of GoTaq Flexi Buffer (5x), 2.5 µl of MgCl_2_, 0.5 µl of dNTPs, 0.125 µl of GoTaq DNA Polymerase, Promega, Madison, WI, in a total reaction volume of 25 µl. The thermocycler program was set to one cycle at 96°C for 4 min, 40 cycles at 96°C for 30 s, 51°C for 30 s, 72°C for 90 s, and one cycle at 72°C for 4 min. The PCR products were subject to electrophoresis in 2% agarose gel with ethidium bromide; a PCR product without DNA template was used as negative control. *Culex pipiens* s.l., *Cx. restuans*, and *Cx. salinarius* were identified according to the amplicon size (698 bp, 506 bp, and 175 bp, respectively) as visualized on the gel, as described by Crabtree *et al.* (1995).

### Mosquitoes Specimens and Morphological Analysis

The mosquitoes (*Cx. pipiens* s.l. and *Cx. restuans*) used for analysis of morphological characters were reared from eggs collected in standing water inside tires in Monona [21.VI.2017, 12.VI.2017, 14.VI.2017], DeForest [21.VI.2017], and Janesville [15.VI.2017], Wisconsin. Larvae were reared individually in tap water in plastic cups and fed dry yeast. The fourth-instar exuviae were collected and slide mounted in Canada Balsam (SERVA Electrophoresis GmbH, Heidelberg, Germany) for confirmation of taxonomic identity, whereas the adults were point mounted and stored dry.

Characters of interest for identification of *Cx. pipiens* s.l. and *Cx. restuans* were determined utilizing these specimens (*n* = 14). The same specimens were used to confirm the mosquitoes of our region were correctly identified by the primers from Crabtree *et al.* (1995).

Additional adult female mosquitoes from field collections were used to confirm the utility of the characters found by the previously discussed morphological analysis (*n* = 99). For each specimen, the taxonomic identity was determined using the morphological characters proposed here, and then a leg sample was removed and encoded to be subjected to a blinded molecular identification utilizing the molecular methods described. These additional mosquitoes were collected from All Saints Cemetery, Des Plaines, IL. [42°03′54.7″N, 87°53′45.8″W; gravid trap; 19.VI.2018, 31.VII.2018, 14.VIII.2018] and from Blaine [45°07′42.96″N, 93°15′02.52″W; CDC trap CO_2_ baited; 10.VII.2018], Anoka [45°12′51.12″N, 93°22′09.12″W; gravid trap; 18.VII.2018], Waconia [44°51′15.12″N, 93°47′46.68″W; CDC trap CO2 baited; 7.VIII.2018], Mendota Heights [44°52′14.16″, 93°11′49.56W″; CDC trap CO_2_ baited; 31.VII.2018], Minnetrista [44°56′47.4″N, 93°42′39.96″W; CDC trap CO_2_ baited; 24.VII.2018], Lauderdale [44°59′22.2″N, 93°11′54.6″W; gravid trap; 15.VIII.2018], St. Paul [44°53′48.84″N, 93°10′08.4″W; CDC trap CO_2_ baited; 24.VII.2018], Shakopee [44°45′45.72″N, 93°31′49.8″W; CDC trap CO_2_ baited; 31.VII.2018], and Cedar Lake [44°34′16.32″N, 93°25′45.12″W; gravid trap; 25.VII.2018], Minnesota.

All specimens were deposited in the Culicidae collection in the School of Veterinary Medicine, Department of Pathobiological Sciences at the University of Wisconsin–Madison.

The nomenclature of characters generally follows Harbach and Knight (1980), except where otherwise indicated. Generic abbreviations follow Reinert (2009).

## Results

### Characters for Morphologically Distinguishing *Cx. pipiens* s.l. From *Cx. restuans*

**Fig. 1.**
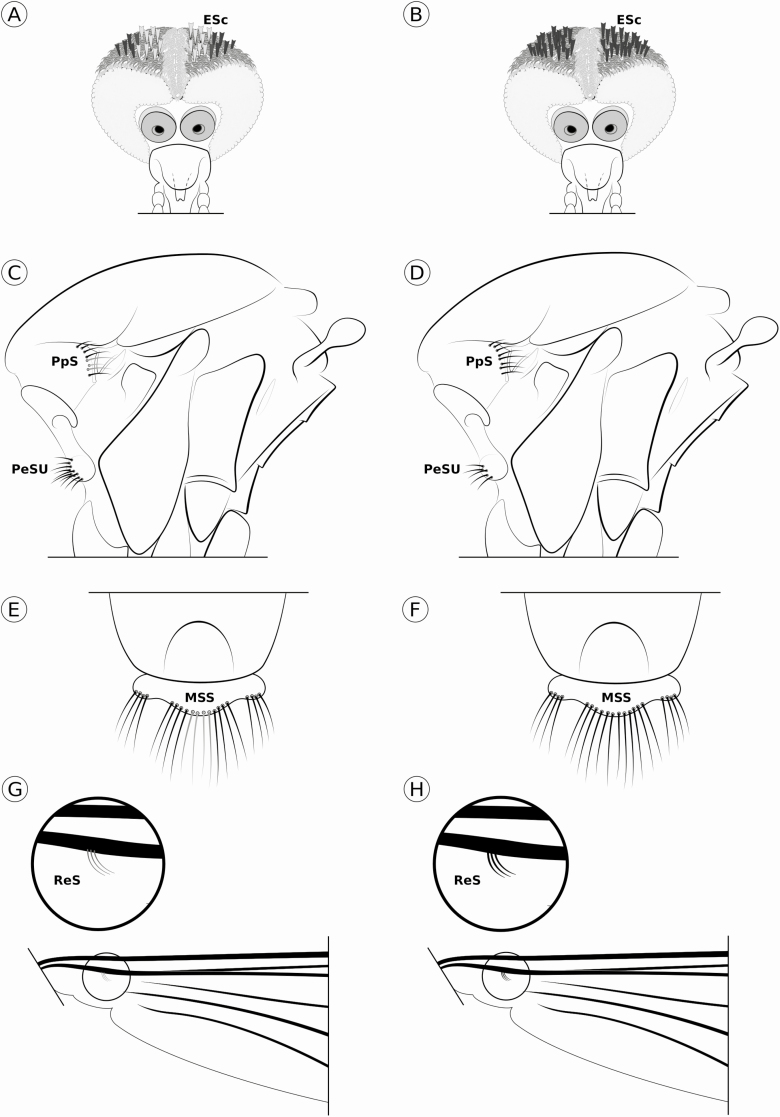
General characters of *Culex pipiens* s.l. (left; A, C, E, and G) and *Culex restuans* (right; B, D, F, and H). A, B: erect scales (ESc) on the dorsal portion of head, dark laterally and pale medially (A) or all dark (B); C, D: lateral view of the thorax, postpronotal setae (PpS) pale/golden, at least one, (C) or all dark (D), upper proepisternal setae (PeSU) six to 12 (C) or four to seven (D); E, F: dorsal view of scutellum, median scutellar setae (MSS) pale/golden, at least one, (E) or all dark (F); G, H: dorsal view of the wing, remigial setae (ReS) thin and pale/golden (G) or thick and dark (H). Check key for additional details.

We uncovered and analyzed five adult female characters that may be of use for the distinction of *Cx. pipiens* s.l. and *Cx. restuans*. We observed that the erect scales on the dorsal area of the head (ESc), distributed from occiput to vertex, were all dark colored in *Cx. restuans* (Fig. 1B); these same scales were, at least medially, pale in *Cx. pipiens* s.l. (Fig. 1A). This is generally consistent with the literature; however, Carpenter and LaCasse (1955/1974) presented this character as ‘usually’ the case for *Cx. pipiens* s.l. We found no evidence to say this is not always the case in our samples. Furthermore, in our samples, these scales (ESc) were more numerous in *Cx. restuans* than in *Cx. pipiens* s.l. (Fig. 1A and B).

The number of setae present in the upper proepisternum (PeSU) on the thorax, as viewed laterally, was useful for separation of *Cx. pipiens* s.l. (six to twelve setae; Fig. 1C), from *Cx. restuans* (four to seven setae; Fig. 1D). These characters have not been described in the literature for *Cx. restuans*. Harbach *et al.* (1985) stated that upper proepisternal setation varies from eight to thirteen setae for the neotype series of *Cx. pipiens*, and Sirivanakarn and White (1978) reported a total of ten to twelve proepisternal setae present in the neotype series of *Cx. quinquefasciatus*.

The coloration of the setae of the postpronotum (PpS) and of the mid lobe of the scutellum (MSS) proved to be useful in our samples as well. For this character, some or all (usually at least one) of the setae of the postpronotum were golden/pale in *Cx. pipiens* s.l. (Fig. 1C), as well as some or all (usually at least one) of the setae of the mid lobe of the scutellum were golden/pale in *Cx. pipiens* s.l. (Fig. 1E); in contrast, all of the setae of the postpronotum and the mid lobe of the scutellum were dark in *Cx. restuans* (Fig. 1D and F). These characters have not been reported in literature.

We also found the wing remigial setae (ReS) may be of some use in the separation of those species, as they appear to be lighter colored and thinner in appearance in *Cx. pipiens* s.l. (Fig. 1G) and very dark and thicker in appearance in *Cx. restuans* (Fig. 1H). Furthermore, we observed three to four remigial setae generally present in *Cx. restuans*, while only two to three setae generally present in *Cx. pipiens* s.l., but overlap proved to occur too frequently to make the seta count useful. To the best of our knowledge, the only account of remigial setae in a description of these species was in the description of the neotype of *Cx. pipiens*, in which Harbach *et al.* (1985) reported the specimen as having two remigial setae.

### Paired Morphological and Molecular Identification of *Culex pipiens* s.l. and *Culex restuans*

To confirm the molecular identification of laboratory reared adults, for which each morphological identification was confirmed with the larval exuvia, DNA was extracted from a single leg and subjected to PCR for the ITS segment using methods and primers from Crabtree *et al.* (1995). To validate these characters in specimens from elsewhere, we acquired and identified 73 adult female specimens to *Cx. pipiens* s.l. and 26 adult female specimens to *Cx. restuans*, from material collected from Des Plaines, Illinois, U.S.A. (50 *Cx. pipiens* s.l., 13 *Cx. restuans*), and from Blaine, Anoka, Waconia, Mendota Heights, Minnetrista, Lauderdale, St. Paul, Shakopee, and Cedar Lake, Minnesota (23 *Cx. pipiens* s.l., 13 *Cx. restuans*) in 2017 and 2018. Each specimen was morphologically identified using the combination of characters described above, then subjected to PCR based identification using the method described in Crabtree et al. (1995). There was 100% agreement between morphological and molecular identification for every specimen tested.

### Paired Morphological and Molecular Identification of *Culex salinarius*

In addition to these findings, we tested and found support for the utility of the presence of short brown scales on the mid lobe of the scutellum on *Cx. salinarius*, as compared to long and *pale scales in Cx. pipiens* s.l. and *Cx. restuans* to distinguish these species (Apperson *et al.* 2002). We observed the only two specimens of *Cx. salinarius* in our collection (UW-Madison Arboretum, Madison, WI) and confirmed the presence of this character state, and PCR for the ITS sequence yielded a product with the correct band size using the primers and methods described by Crabtree *et al.* (1995). We note that in our limited experience with *Cx. salinarius*, the character described by Apperson *et al.* (2002) presents itself more as bright and metallic in color than dark brown *per se*, so we refer to it as ‘copper-colored’.

## Discussion

In the process of assessing these specimens and character states, we also had the opportunity to assess the characters previously used to distinguish these species using existing dichotomous keys. Our experience with characters related to the coloration and shape of the abdominal tergite basal ‘pale bands’ corroborated that of Apperson et al. and Harrington and Poulson, who noted that the character state overlaps in specimens and is of little use for separation of said species (2002, 2008). Based on specimens observed from Wisconsin, Illinois, and Minnesota, we likewise observed that abdominal tergite scaling pattern is not useful for identification of these two species because 1) there is significant overlap of the character between the species such that some specimens of both species present the character as reduced in some or most tergites, thereby forming a very thin ‘band’ or no ‘band’, and 2) because of potential confusion with other *Culex* species if this is the character on which the observer is focused.

Furthermore, we did not find that thoracic integument coloring was useful for identification of these mosquitoes from this region. The use of this character is not consistent in the literature, with most modern Nearctic keys attributing the ‘reddish brown’ character as exclusive to *Culex erythrothorax* (e.g., Carpenter and LaCasse 1955/1974; Darsie Jr and Ward 1981, 2005). The same can be said of the character of scutal scaling. We did find that the scutal scales are dark brown and short in *Cx. restuans*, as compared to bright yellow and rather long (giving the impression the scutum scaling to be quite ‘shaggy’) in *Cx. pipiens* s.l.; however, the shape of the scales is not well described as ‘not falcate’ in *Cx. restuans,* as reported by Apperson *et al.* (2002). The scutal scales of *Cx. restuans* in our sample seemed to be falcate, but not dorso-ventrally expanded by a significant amount, which gives them a more ‘hair-like’ appearance at a glance, rather than the more archetypal falcate scales present in *Cx. pipiens* s.l. This character may furthermore be misinterpreted as the ‘hair-like’ scales of *Cx. erythrothorax* as presented in the keys of Carpenter and LaCasse (1955/1974) and Darsie Jr and Ward (1981, 2005), resulting in a misidentification. Additionally, *Cx. salinarius* observed herein from Wisconsin also presented the character of short brown scutal scales, with lack of significant dorsovental expansion, giving a more ‘hair-like’ appearance than the ones of *Cx. pipiens* s.l.

In summary, *Cx. pipiens* s.l. and *Cx. restuans* are morphologically differentiable, but existing keys do not sufficiently delineate the morphological differences between these two species to provide a reliable identification. The combination of characters described herein may be of use to ascertain the correct identification of a few *Culex* species that currently have equivocal characters in available keys. Should the user identify *Cx. erythrothorax*, *Cx. interrogator*, *Cx. chidesteri*, *Cx. salinarius*, *Cx. pipiens* s.l., or *Cx. restuans* using existing keys, the characters below should provide a degree of certainty to the identification. The following key was derived from characters present in the literature and those presented in this paper. The key should be taken as provisional until these characters have been thoroughly vetted for specimens of these species from different regions of the Nearctic or, preferably, the American continent as a whole. Additional research is needed to further increase our knowledge of the morphological traits of all these species, and also to improve and update reference material (both keys and descriptions) for the Nearctic mosquito fauna.

### Key to Adult Female *Culex* (*Culex*) (in Part)

The purpose of this key is to provide a way to confirm the identities of certain *Culex* (*Culex*) species that have been mischaracterized in reference material or have not been adequately separated in previous keys. More specifically, we recommend the consultation of the following key if the reader arrives at the identification of *Cx. erythrothorax*, *Cx. interrogator*, *Cx. chidesteri*, *Cx. salinarius*, *Cx. pipiens* s.l., or *Cx. restuans* by utilizing current keys to Nearctic *Culex* species. We believe this provides a more accurate identification, at least within Midwestern states in the United States, with the limit of its usage confined to the Nearctic region, due to the likely variation in the character states of Neotropical *Cx. restuans* in relation to the Nearctic.

1. Abdominal tergites VIII and most of VII pale scaled, other tergites bearing narrow basal pale bands, sometimes indistinct; abdominal sternites covered by pale scaling only; base of mid lobe of scutellum with short ‘copper colored’ scales or with long pale scales .................................................................... 2
Abdominal tergites with basal pale bands, usually rather broad, rarely very narrow to indistinct; abdominal sternites mostly covered by pale scaling, usually with dark scales intermixed; base of mid lobe of scutellum with long pale scales .................................................................................................. 3
2(1). base of mid lobe of scutellum with long pale scales .............................................................................. *Cx. erythrothorax*
base of mid lobe of scutellum with short “copper colored” scales; tergites usually with a few pale scales scattered at apex ........................................................................... *Cx. salinarius*
3(1). Postspiracular patch of scales usually present; very narrow pale rings at joint of tasomeres usually present; erect scales of dorsum of head dark ...................................................................................................................... *Cx. chidesteri*
Postspiracular patch of scales absent; joints of tarsomeres usually without pale markings; erect scales of dorsum of head dark or pale ones medially .................................................... 4
4(3). Wing vein R_2 + 3_ about one third of the length of wing cell R_2_, erect scales of dorsum of head dark ......... *Cx. interrogator*
Wing vein R_2 + 3_ about one sixth or about one fourth the length of wing cell R_2_, if one fourth, then erect scales of dorsum of head pale medially ............................................................ 5
5(4). Middorsocentral patches of pale scaling present or absent; erect scales of dorsum of head dark (Fig. 1B); upper proepisternum with four to seven setae (Fig. 1D); postpronotum with all. setae dark (Fig. 1D); mid lobe of scutellum with all setae dark (Fig. 1F); remigial setae thick, dark, three to four (usually three) (Fig. 1H) ............................................................................ *Cx. restuans*
Middorsocentral patches of pale scaling absent; erect scales of dorsum of head pale medially, others dark (Fig. 1A); upper proepisternum with six to 12 setae (Fig. 1C); postpronotum usually with at least one seta pale/golden (Fig. 1C); mid lobe of scutellum with at least one seta pale/golden (Fig. 1E); remigial setae thin, pale, two to three (usually three) (Fig. 1G) ........................................................................................ *Cx. pipiens* s.l.
